# A learning-based tip contact force estimation method for tendon-driven continuum manipulator

**DOI:** 10.1038/s41598-021-97003-1

**Published:** 2021-09-01

**Authors:** Fan Feng, Wuzhou Hong, Le Xie

**Affiliations:** 1 Institute of Forming Technology & Equipment, Shanghai Jiao Tong University, Shanghai, 200030 China; 2 Institute of Medical Robotics, Shanghai Jiao Tong University, Shanghai, 200240 China

**Keywords:** Electrical and electronic engineering, Engineering, Biomedical engineering

## Abstract

Although tendon-driven continuum manipulators have been extensively researched, how to realize tip contact force sensing in a more general and efficient way without increasing the diameter is still a challenge. Rather than use a complex modeling approach, this paper proposes a general tip contact force-sensing method based on a recurrent neural network that takes the tendons’ position and tension as the input of a recurrent neural network and the tip contact force of the continuum manipulator as the output and fits this static model by means of machine learning so that it may be used as a real-time contact force estimator. We also designed and built a corresponding three-degree-of-freedom contact force data acquisition platform based on the structure of a continuum manipulator designed in our previous studies. After obtaining training data, we built and compared the performances of a multi-layer perceptron-based contact force estimator as a baseline and three typical recurrent neural network-based contact force estimators through TensorFlow framework to verify the feasibility of this method. We also proposed a manually decoupled sub-estimators algorithm and evaluated the advantages and disadvantages of those two methods.

## Introduction

Continuum manipulators (CMs) have received a great deal of attention in industry^1,2^, especially in robot-assisted surgery^3^ , due to their higher dexterity and safety when compared to rigid-link manipulators. A tendon-driven mechanism is the most commonly used in the field of surgical robotics. However, force-sensing technology is one of the major limitations in the development of surgical robots, because in master-slave surgical robotic systems that lack force feedback, the operator can rely on only visual feedback, such as the deformation of tissue under load to estimate the contact force^4^. This approach is highly subjective and influenced by the experience of the surgeon, which poses an added risk for robot-assisted surgery. The need for force feedback in robot-assisted surgery has led to a series of studies on force estimation methods for continuum manipulators.

Using strain information is the most common and straightforward method for the deflection sensing of flexible continuum manipulators^5^. For instance, Karthikeyan et al.^6^ developed an S-shaped force sensor; the shape and positioning of the sensor on the instrument was finalized using finite element analysis. Noh et al.^7^ developed a contact force sensor based on three dyadic S-shaped beams and three optoelectronic sensors for the CM. Through calibration with finite element analysis, the sensor can measure the two-dimensional tip contact force of the continuum manipulator.

In recent years, there has been an increasing number of studies on the use of fiber Bragg grating (FBG) sensors for shape or force sensing. Most researchers have adopted the scheme of distributing three optical fibers evenly around the CM^8–12^. However, a FBG is susceptible to temperature, which needs to be considered in the static model. In addition, a wavelength shift in the FBG will also be affected in the case of small radius bending^13^, which will undoubtedly limit the maximum bending curvature of the CMs.

With the development of deep learning techniques, there are also researchers that use machine vision approaches to achieve force sensing for CMs. Su et al.^14^ and Haouchine et al.^15^ both present methods for vision-based force estimation in surgical robotic systems by detecting tissue deformation. However, these machine vision approaches require more computational resources and have a lower execution rate without GPU acceleration.

For the above force estimation solutions, all require the addition of channels in the flexible deformation part of the CM or external sensing devices. This will undoubtedly lead to an increase in the diameter of the CM, which in some applications where the size is strictly constrained, such as robotic surgery in the ear, nose, and throat, may impose a significant limitation on its dexterity^3^. In addition, these methods also increase the complexity of the mechanical structure and may cause greater errors in the modeling of the CMs.

Interestingly, the tension of the driving tendon, which is important information in a tendon-driven continuum manipulator, is often overlooked, and studies on force estimation using this information are scarce. Gao et al.^16^ designed a strategy to use a FBG as a tendon for simultaneously driving and measuring the force. Back et al.^17^ designed a force feedback method on a catheter by using a kinematic model as well as strain gauges to detect tension. These strategies still require additional pose sensors for the global tip pose of the CM. Bajo et al.^18^ designed a hybrid motion/force control system by using a static model of the CM, but it was based on extensive theoretical modeling and analysis work. It still takes a great deal of time and resources to analyze, calculate, and calibrate kinematic and static models that are based on the mechanical structure of a CM.

Rather than the complex modeling, Goldman et al.^19^ used the support vector machine to capture the friction and correct the uncertainties of the CM. However, this method still relied on elaborated analysis and modeling of the mechanics of the CM. Compared to this, Braganza et al.^20^ used artificial neural network (ANN) algorithms to compensating for the nonlinear uncertain dynamics of the CM. In addition to this, many scholars have used ANN algorithms for the kinematic modeling^21–24^, shape estimation^25^, adaptive neural network control^26^ of a CM. Although the ANN-based modeling approach relies more on training data, it can take into account the manufacturing and assembly errors of the CM, the period of tendon contracting or stretching, and so on. Therefore, it can achieve higher modeling efficiency and accuracy than by building an analytic kinematic or static model^27^. Li et al.^28,29^ used proximal-end measurements as input and distal-end tendon force as output in tendon-sheath mechanisms that applied a multi-layer perceptron (MLP) and recurrent neural network (RNN) algorithms. Jakes et al.^30^ has done a similar work which using a RNN as the tension predictor. The motor positions were considered to be continuous control input signal to predict the internal tension. But additional modeling and calibration steps are still required to estimate tip force from the predicted tension.

From the existing studies mentioned above, it appears that modeling using tension information is a feasible approach to realize tip contact force estimation without increasing the diameter of a CM for robot-assisted surgery. However, a extensive and accurate modeling effort is required for a specific CM and the model is not generally applicable among different structure of CMs. The loading paths for master-slave tendon-driven CMs with two degrees of freedom (DOF) are more complex, and the estimation of the tip contact force based on learning method directly from the tensions and motor positions has still not been studied.

In that case, we applied a learning-based modeling method for the contact force estimator of tendon-driven continuum manipulators without requiring sensors at the body of it. In this method, we propose to use both the tension and displacement of the tendons as neural network inputs to determine the actual 2-dimensional tip contact force of the CM in real time by a pre-trained RNN. This method does not increase the diameter of the CM and is not theoretically limited to the structure used in this paper. It can also be used in the tip contact force estimation for tendon-driven CMs of other structures, even soft manipulators. At the same time, this learning-based approach does not require extensive and accurate modeling work, and can be used as a force estimator for a specific CM structure after the training samples have been collected and the neural network has been trained. This method is of practical value for the CMs that used in robot-assisted surgery where its size are strictly constrained.

In “Design and methods” Section, the principles and design steps of such a method are described in detail. In “Experiments” Section, a training data acquisition platform is built. A MLP as a baseline and typical RNNs were trained simultaneously, and the accuracy and dynamic performances of each were compared to verify the effectiveness of this method. In addition, a manually decoupled sub-estimators algorithm with lower time complexity is also proposed. “Conclusions and future work” Section summarizes this approach and illustrates our future work based on it.

## Design and methods

### Problem description

**Figure 1:**
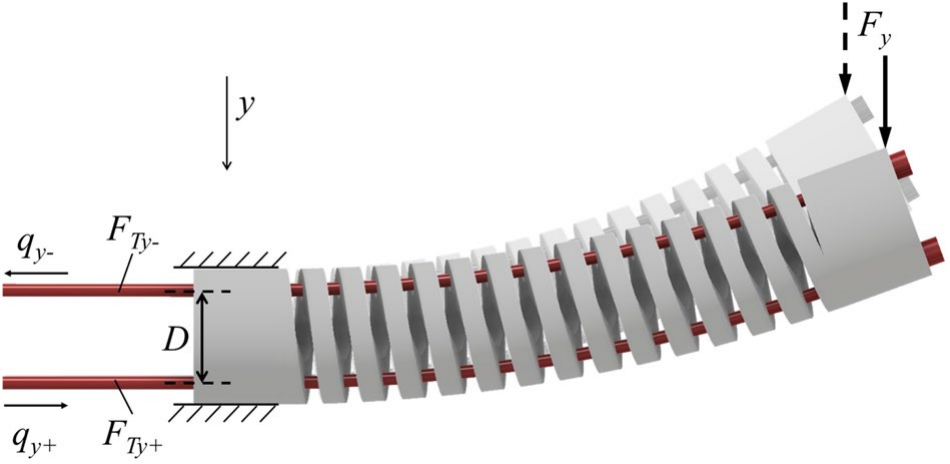
Tendon-driven helical continuum manipulator subjected to a unidirectional contact force $F_y$.

As shown in Fig. 1, our previous study^31^ proposed the structural design of a tendon-driven flexible helical joint for a CM, in which we also performed its kinematics and static modeling. The functional relation of the tendons’ displacements and tensions to the deflection angle of the flexible joint was obtained as follows:

1 $$\begin{aligned} \theta = \frac{{2\Delta q_{y-}}}{D} = \frac{{\pi {\lambda _\theta }N{D^2}{F_{Ty-}}}}{4}\left( {\frac{1}{{EI}} + \frac{1}{{GJ}}} \right) \end{aligned}$$

where *E* and *G* are the modulus and shear modulus of elasticity, respectively. *I* and *J* are the second moment and polar moment of the cross section of the coil part, respectively. *N* is the total number of coils of the helical structure. *D* is the mean coil diameter of the flexible joint. ${F_{Ty-}}$ is the pulling force. ${\lambda _\theta }$ is the compensation factor. From the kinematics and static model, it is found that the displacement of tendon $\Delta q_{y-}$ and the pulling force ${F_{Ty-}}$ should be positively proportional as follows:

2 $$\begin{aligned} \Delta q_{y-} = \left[ {\frac{{\pi {\lambda _\theta }N{D^3}}}{8}\left( {\frac{1}{{EI}} + \frac{1}{{GJ}}} \right) } \right] {F_{Ty-}} \end{aligned}$$

Equation (2) indicates that in the ideal case and in the absence of external forces on the tip the of CM, the displacement of the tendon should have a linear relation with its tension. As shown in Fig. 1, for example, in the state without an external force, ${F_{Ty - }}$ and ${F_{Ty + }}$ shall be fixed when ${q_{y - }}$ and ${q_{y + }}$ reach a certain position after being driven by servomotors. Both ${q_{y - }}$ and ${q_{y + }}$ can be obtained by monitoring the encoders, while ${F_{Ty - }}$ and ${F_{Ty + }}$ can be obtained by tension sensors connected in series with the tendons. However, when the tip of the CM is subjected to a unidirectional contact force ${F_{y}}$, the values of ${q_{y - }}$ and ${q_{y + }}$ obtained by the encoders will do not change, but the tensions ${F_{Ty - }}$ and ${F_{Ty + }}$ will change depending on the magnitude of ${F_{y}}$, yielding $\Delta {F_{Ty - }}$ and $\Delta {F_{Ty + }}$. In the ideal case, this relationship can be characterized by a specific functional relation *g* as follows:

3 $$\begin{aligned} {F_{y-ideal}} = g\left( {{q_{y + }},{q_{y - }},\Delta {F_{Ty + }},\Delta {F_{Ty - }}} \right) \end{aligned}$$

It is possible to describe the function relation *g* by developing a static model. However, in reality, the manufacturing and assembly error of the CM cannot be well modeled [21], but this systematic error also satisfies a specific relation $\varphi$ as shown in the following, where $\varepsilon$ is an unpredictable random term:

4 $$\begin{aligned} \begin{aligned} {F_y} =&g\left( {{q_{y + }},{q_{y - }},\Delta {F_{Ty + }},\Delta {F_{Ty - }}} \right) +\\&\varphi \left( {{q_{y + }},{q_{y - }},{F_{Ty + }},{F_{Ty - }}} \right) + \varepsilon \end{aligned} \end{aligned}$$

**Figure 2:**
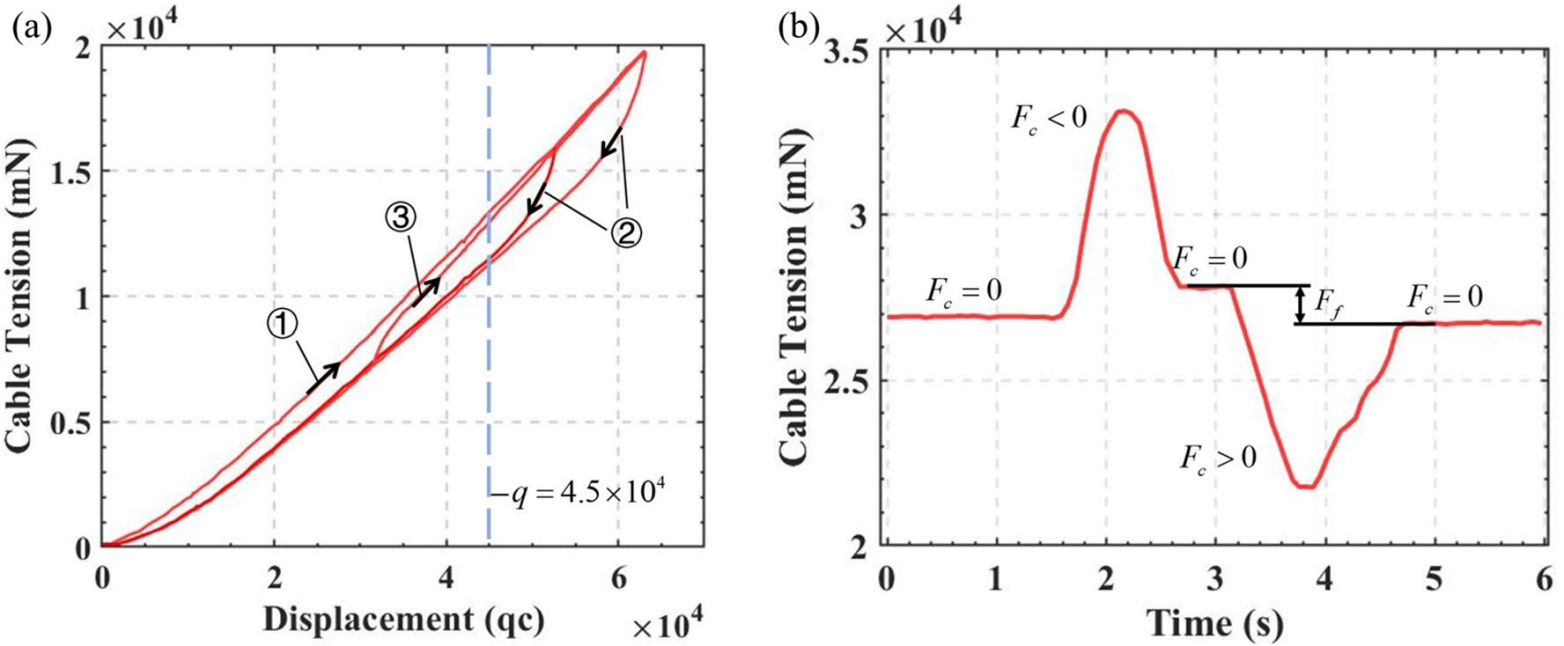
(**a**) Three loading paths of a tendon-driven CM. (**b**) Path of tension changes influenced by interface friction.

For a rigid-link manipulator, the robot starts motion from the current position, and in most applications, it then moves on a trajectory on a sequential point path. The inclusion of the current joint configuration in the artificial neural network has a positive effect on the estimation of joint angles for the next desired position^32^. However, in the case of a tendon-driven CM, not only the current joint configuration but also information from several even earlier paths have a strong influence on the next desired position due to the influence of nonlinear frictional forces on the tendons. For example, as shown in Fig. 2a, considering only the case where a single tendon is activated in one direction, the tension on it may have three states at the displacement of $q = 4.5 \times {10^4}~\mathrm {qc}$: $\textcircled {\small {1}}$ a pulling phase, $\textcircled {\small {2}}$ a releasing phase, and $\textcircled {\small {3}}$ a pulling after releasing phase. The shape of the curve varies depending on the starting point. This leads to an infinite number of possibilities for the corresponding tension when the tendon is at a particular position if no loading path feature is considered.

In addition, the surface state of the tendons and the assembly scheme have a great influence on the interface friction between the CM and the tendons. As shown in Fig. 2b, when the CM is loaded to a position from the state without an external force in the path $\textcircled {\small {1}}$ and then a contact force ${F_c} < 0$ is applied, the tension becomes larger. However, the tension will not be the same as that at $t=1~\mathrm {s}$, when the ${F_c}$ returned to 0. Similarly, if the contact force ${F_c} > 0$ is applied now and then returns to 0, the tension will neither return to the values at $t=1~\mathrm {s}$ or $t=3~\mathrm {s}$. In the range $F_f$ marked in Fig. 2b, it is not possible to determine the magnitude of the tip contact force without considering the path of the tension changes.

Therefore, without considering the extraction of the loading path and tension variation path features through complex model analysis, in order to better process sequence information, simple recurrent networks (SRN), long short-term memory (LSTM) networks, and gated recurrent unit (GRU) networks are better able to take into account the loading path features for sequence learning. Data processed by an RNN can then be fed into a subsequent neural network, such as the fully connected layers, to perform prediction and other operations. It is worth noting that its generalization ability across devices can be adapted by the learning process. That is, although the different structures of different CMs may lead to large differences in their systematic errors $\varphi$, learning-based methods can fit systematic errors in their respective sample collection and training process.

### Contact force estimator

If the contact force is considered as a 2-dimensional vector, a total of four tendons can achieve the 2-DOF movement of the CM. Consider the state without an external force as part of the mechanical properties of the system as well. In the kinematic model of the CM, the two tendons that control the same direction have a specific position relationship, which means ${q_{x + }} + {q_{x - }} = {q_{y + }} + {q_{y - }} = 0$. Therefore, the tendons’ displacement can be expressed as $q = \left[ {{q_{x + }},{q_{y + }}} \right] \in {\mathbb {Z}^2}$. The tensions can be expressed as ${F_T} = \left[ {{F_{Tx + }},{F_{Tx - }},{F_{Ty + }},{F_{Ty - }}} \right] \in {\mathbb {R}^4}$. The tip contact force of the CM can be expressed as ${F_c} = \left[ {{F_x},{F_y}} \right] \in {\mathbb {R}^2}$. Assuming that the information from the current moment *t* and its past total of *n* moments can be used to accurately determine the loading path and the tensions’ changing path, then the number of holds is determined as follows:

5 $$\begin{aligned} \begin{aligned} {\widehat{{F_c}}_t} = f\left( {{q_t},{F_{Tt}},{q_{t - 1}},...,{q_{t - n + 1}},{F_{T(t - n + 1)}}} \right) \end{aligned} \end{aligned}$$

where ${\widehat{{F_c}}_t}$ is an estimated value of the contact forces at moment *t*. Our goal is to fit the functional relation *f* in (5) using enough data for the training of the RNN. It is worth noting that *f* not only fits the CM static properties *g* in (4) but also fits the systematic errors, such as the manufacturing and assembly errors of the CM ($\varphi$) at same time, thus achieving a higher accuracy than the static modeling method.

**Figure 3:**
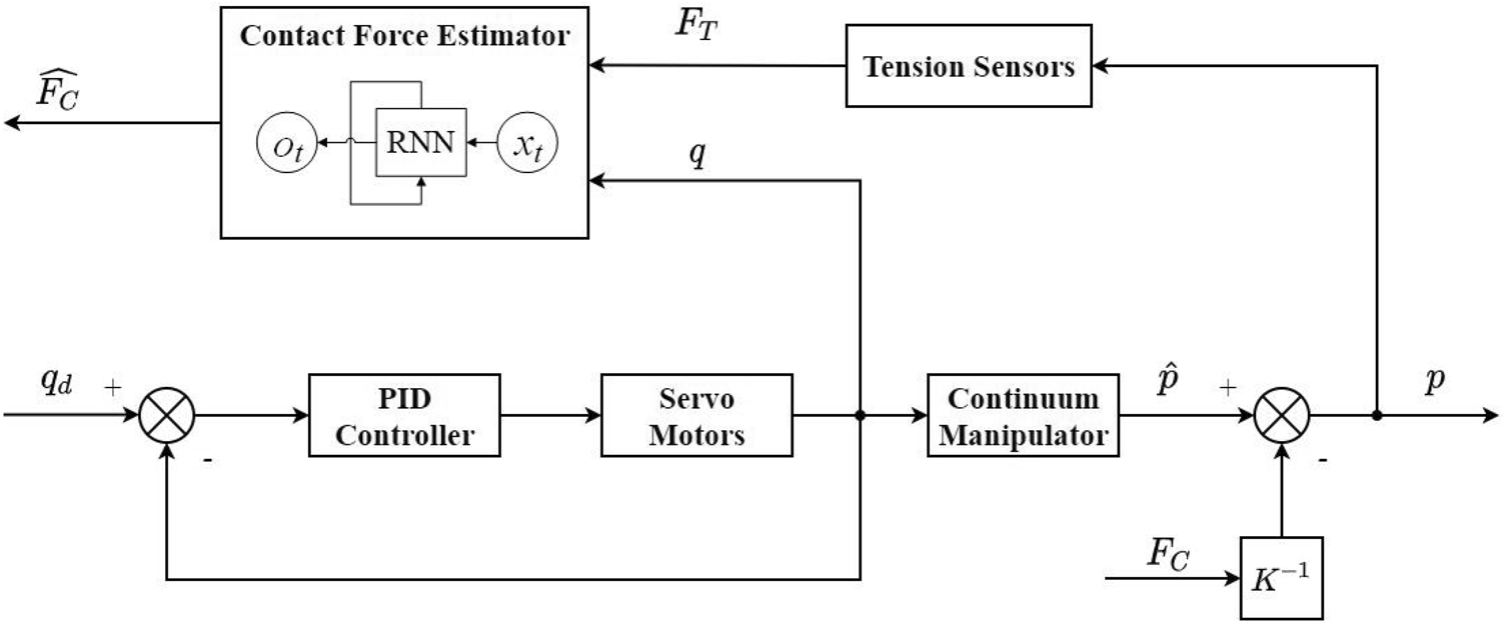
Design of the real-time contact force estimator.

Once the network weights of the contact force estimator have been trained, the real-time contact force estimation of the CM can be achieved using the algorithm block diagram shown in Fig. 3, where ${q_d}$ is the desired position of the servomotor, and ${\widehat{p}}$ is the tip position of the CM without an external force. When the CM’s tip is subjected to a contact force ${F_c}$, the CM’s tip position will be shifted to *p*. The specific offset is determined by the unknown stiffness matrix *K*. The contract force estimated at the current moment ${\widehat{{F_c}}}$ can be calculated in real-time by taking the current moment and the past *n* moments of the tension sensors’ and servomotor encoders’ data as inputs to the RNN.

## Experiments

### Experimental setup

**Figure 4:**
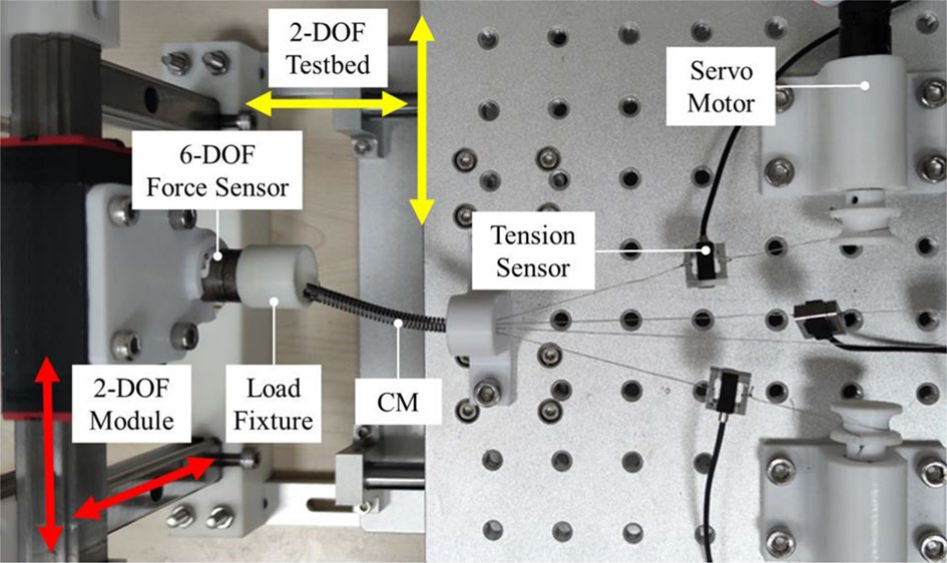
Training data acquisition platform.

Based on the above theoretical analysis, in order to implement this contact force estimator, the acquisition of training data for the RNN should first be completed. For this purpose, we designed a data acquisition platform as shown in Fig. 4 for the CM’s tip contact forces testing. The CM’s flexible joint outer diameter was 5.0 mm, and the tendons were steel wires with a 0.3 mm diameter, which were connected and driven by RE16 servo motors (Maxon Motor AG, Switzerland) with 370:1 reduction ratio planetary gearheads. A DYLY-109 tension sensor (Da Yang Sensing System Engineering, China) was installed in series with each tendon, which could output a voltage (0–5 V) with a weight transmitter. The EPOS2 24/2 controller’s (Maxon Motor AG, Switzerland) 12-bit AD acquisition port was used for the real-time monitoring of those voltages. One of the EPOS2 controllers was connected to the PC via USB, when they were communicating with each other by CANOpen. These were installed on a 2-DOF testbed. At the same time, a 2-DOF contact force acquisition module was mounted on the front of the testbed. A Nano17 (ATI Industrial Automation, USA) 6-DOF force sensor is connected to the PC via a PCI-6220 data acquisition board (National Instruments, USA). A load fixture was attached to the Nano17 sensor to modify the measurement of the 2-DOF contact force. Those devices enabled the real-time monitoring and acquisition of the motor encoders, tensions, and contact force, which were all simultaneously recorded at 50 Hz. In the PC, those data were used as neural network training input and labels, respectively.

Due to the small displacement (less than 10 mm) of the tendons, sufficient space was left between the tension sensors such that no interference occurs. It is worth stating that the absolute accuracy of the tension sensors is not strictly necessary, but the relative accuracy. Because the errors caused by absolute accuracy can be considered as a part of systematic error which will be compensated by machine learning methods.

In the data acquisition phase, the tip of the CM was first placed in the load fixture, and then the servo motors were driven randomly to control the tip position of the CM and position of the 2-DOF force sensor module at the same time to make random touching for generating contact forces. This process was repeated until tens of thousands of valid data are obtained.

### Learning overview

**Figure 5:**
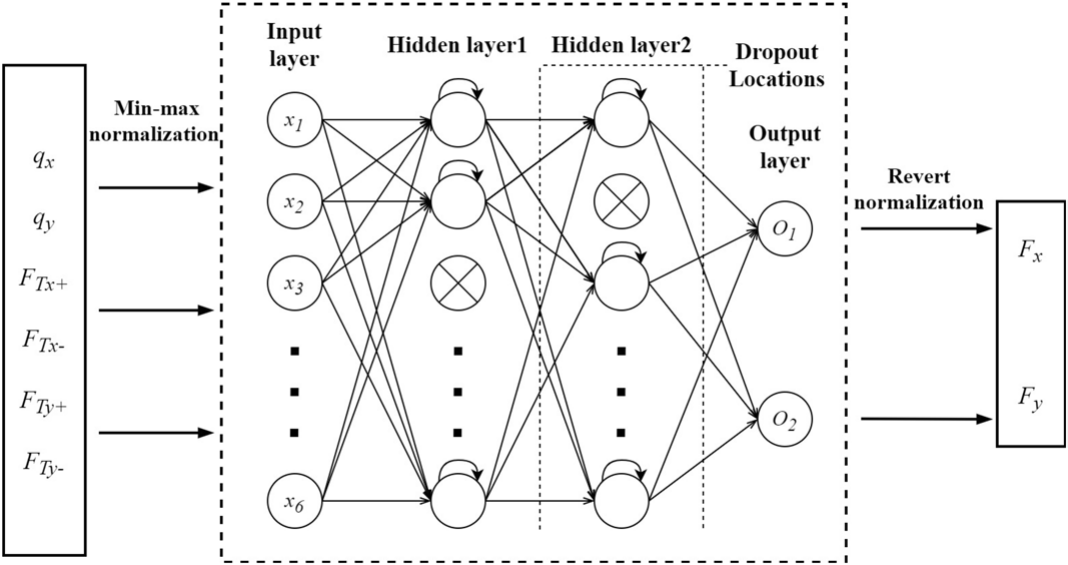
Overview of the RNN-based contact force estimator.

After acquiring the data from the training data acquisition platform, the data were normalized by min-max scaling, and then TensorFlow 2.3.0 was used to build and train the MLP, SRN, LSTM, and GRU neural networks. The number of neurons in the first recurrent layer was 80, and the number of neurons in the second recurrent layer was 100. Both layers used a dropout rate of 20% to prevent the neural networks from overfitting^33^. Since the output value was the tip contact force of the CM, $[ {{F_x},{F_y}} ] \in {\mathbb {R}^2}$, there were two neurons in the fully connected layer (Fig. 5). The training method was configured to use the Adam optimizer with a learning rate $\alpha =0.001$ and exponential decay rates for the moment estimates of $\beta _1 =0.9$ and $\beta _2 =0.999$, as default^34^. To reduce the impact of outliers on the model, we used Huber loss as the loss function with $\delta = 1.0$, since some manual operations were involved in the training data collection, meaning some data anomalies were unavoidable. Meanwhile, a MLP with the same number of neurons in the two hidden layers is also used as a baseline for comparison. Rectified linear unit (ReLU) is used as the activation function for each neuron of the MLP.

**Figure 6:**
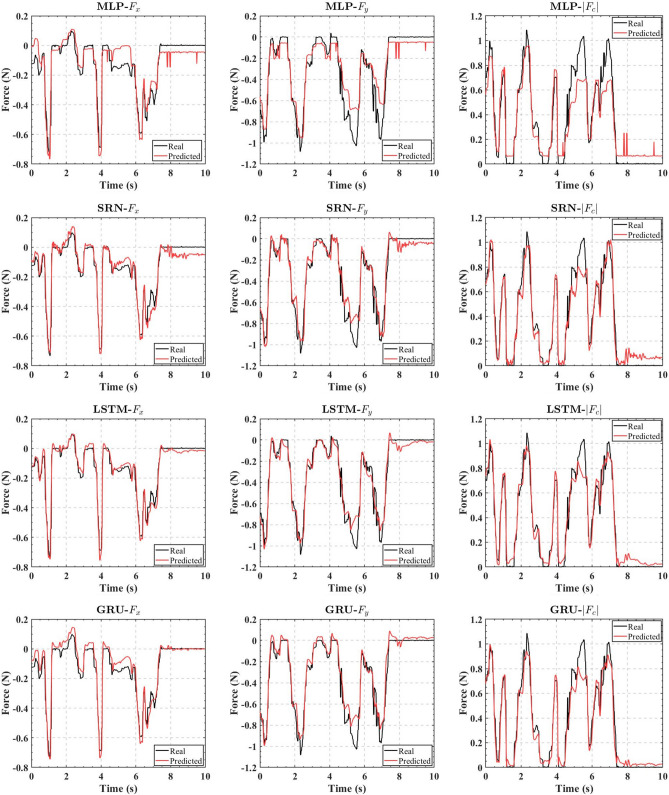
Accuracy of the ANN-based estimators for $F_x$, $F_y$ and $|F_c|$.

The data set was divided into a training set and a validation set, and the training set with 22 850 valid data was sent to the RNN for training. The batch size was 64, and a total of 100 epochs were trained. A random 10-s interval of data was taken as the validation set. And the performance of the MLP and the three trained RNNs on the validation set is shown in Fig. 6.

**Figure 7:**
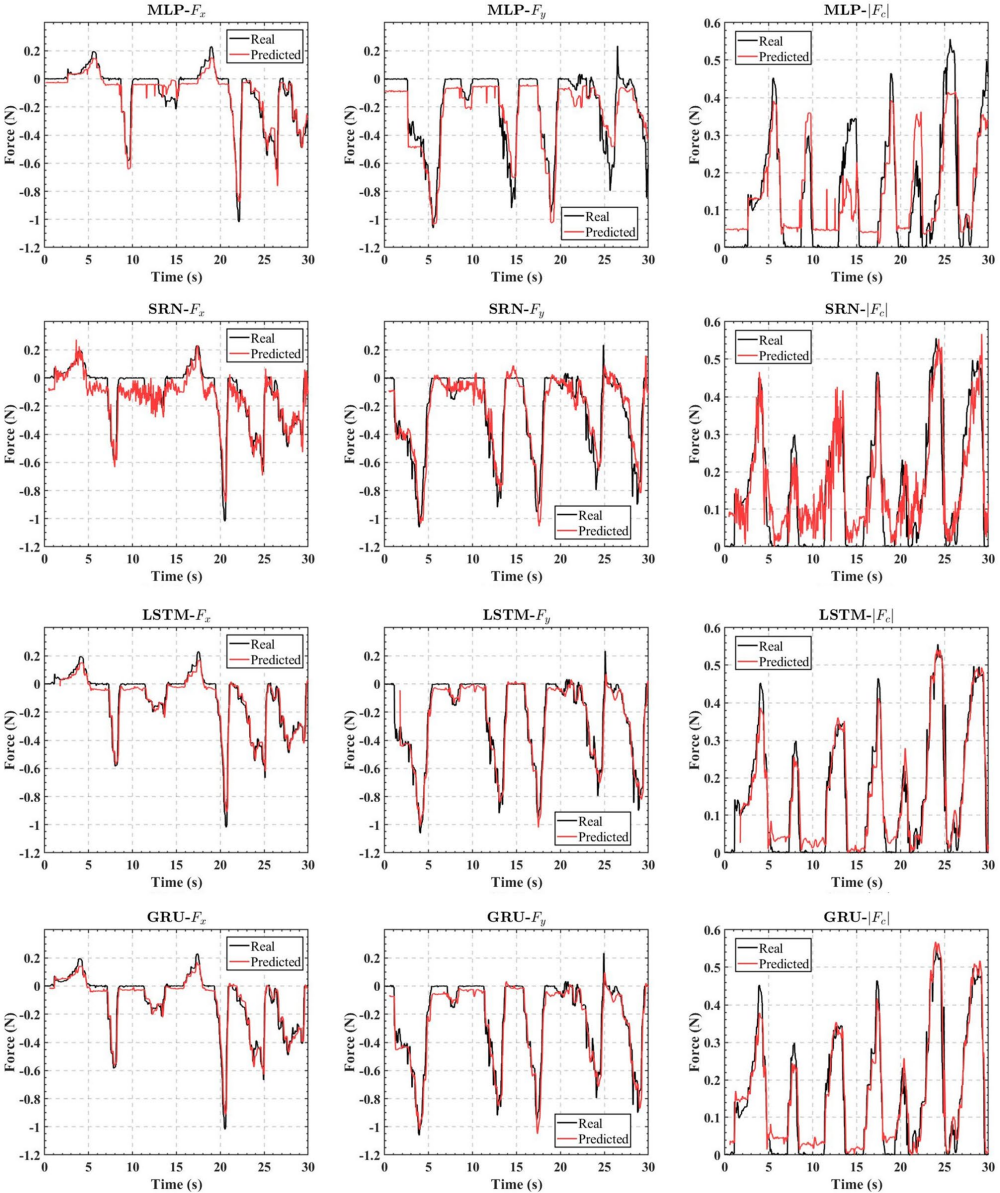
Dynamic performances of the real-time force estimators.

In order to test the dynamic performances of the real-time contact force estimators, we built those estimators with the algorithm shown in Fig. 3 using the same experimental platform. Different from the data acquisition phase, several learning-based contact force estimators were deployed in their own separate threads for real-time estimation and were recorded simultaneously with the contact force data of 6-DOF force sensor as the ground truth. Since the input shape should be a three-dimensional array with 1 sample, 50 time steps, and 6 feature, the force estimator was activated after the first 50 data were collected, which were then fed into the pre-trained ANN, and those 50 data were continuously updated in subsequent sampling sessions to achieve the real-time output of the estimated contact force. The dynamic performances of those real-time estimators in a 30-s interval are shown as examples in Fig. 7. We can see that MLP-based estimator has the worst accuracy; SRN-based estimator has larger fluctuations in predicted values; LSTM-based estimator and GRU-based estimator have a similar accuracy. Those estimators do not have a significant delay for the real-time estimation.

In order to evaluate the accuracy of the model, the root mean square error (RMSE) and the mean absolute error (MAE) were given. However, although the SRN, LSTM and GRU networks all achieved good results in the validation set, the execution time cost should also be evaluated for a real-time force estimation system. For the computational speed of the RNN also affected the dynamic performance of the estimator. So the above trained networks were predicted 2000 times each, and their computation times were recorded. The averages of these computation times are presented in Table 1 to evaluate the delay performance. The CPU version of TensorFlow 2.3.0 was used in the deployment of the ANN due to the small size of the network used and the advantage of the CPU for processing sequential operations. Our PC configuration was as follows: AMD Ryzen 7 3700X 8-Core processor and 16 GB of RAM.

**Table 1:** A summary of estimator performance.

ANN Structure	RMSE, MAE of $F_x$ (N)	RMSE, MAE of $F_y$ (N)	RMSE, MAE of $\left| {{F_c}} \right|$ (N)	Execution time cost
MLP	0.072, 0.049	0.121, 0.071	0.127, 0.083	26.5 ms
SRN	0.047, 0.038	0.088, 0.033	0.089, 0.027	28.0 ms
LSTM	0.037, 0.021	0.077, 0.030	0.076, 0.030	29.6 ms
GRU	0.048, 0.031	0.084, 0.034	0.085, 0.041	29.1 ms

**Figure 8:**
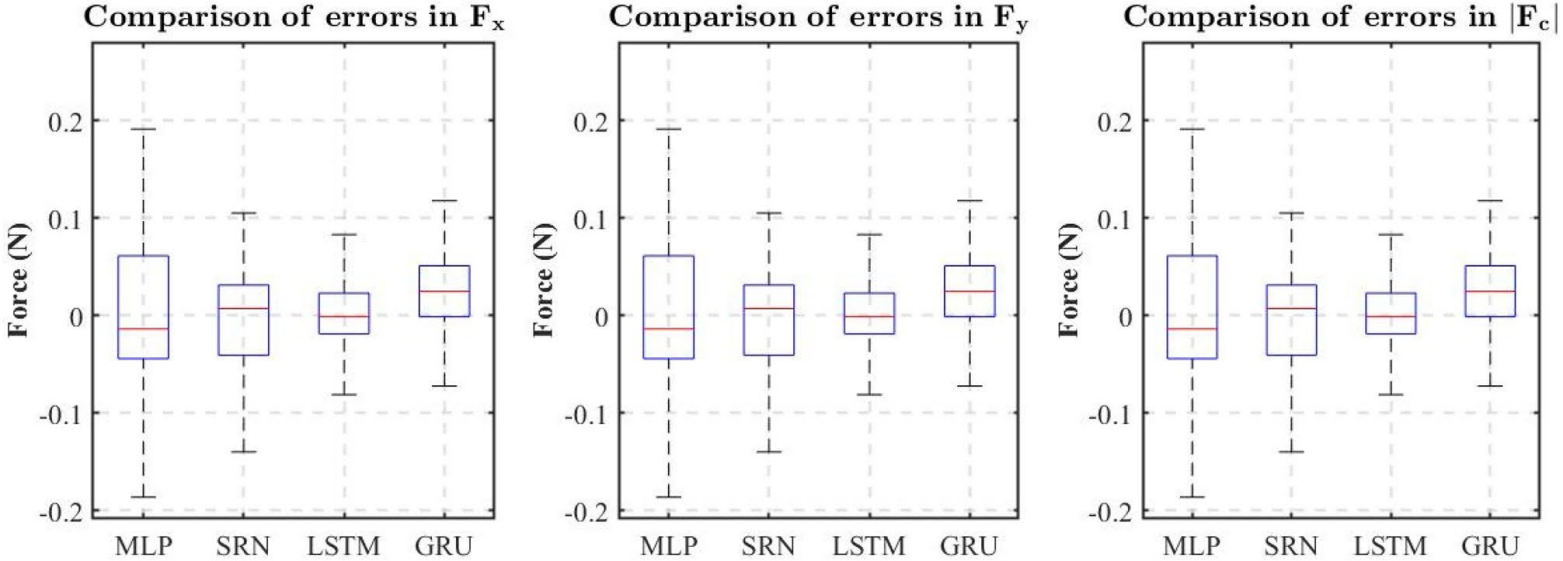
Comparison of the errors of each force estimator.

From Table 1, it can be seen from RMSEs that the RNN of all three structures reached an accuracy of less than 0.05 N for $F_x$ and less than 0.09 N for both $F_y$ and $\left| {{F_c}} \right|$. Table 1 also shows that the MAE of errors are significantly smaller than the RMSE of errors, which means that there are some large values in the errors data. Figure 8 shows the box plot for each force estimator, which more clearly represents the difference in performance of each force estimator. From Fig. 8, we can learn that most of the error values are lower than 0.05 N, but there are still a few error values over 0.1 N, which is the reason why the MAE is lower than the RMSE.

The performance differences in the three RNN-based force estimators are not significant. On the contrary, the MLP-based force estimator exhibits worse accuracy due to the inability to take into account the time series information. This leads to a corresponding insensitivity of the force estimator under the influence of a small contact force that the static friction can not be overcome, for example, the MLP-based estimator for *x*-direction at $t=4.5~\mathrm {s}$ shown in Fig. 6. And due to the static friction, it is difficult to obtain a correct force value when the contact force returns to zero.

### Learning with manual decoupling

**Figure 9:**
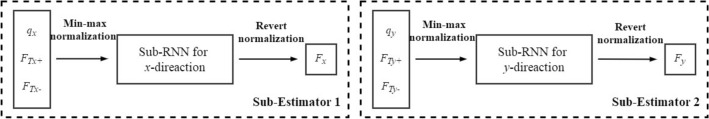
Manual decoupling with sub-estimators for two directions.

For a 2-DOF CM driven by four equally distributed tendons, two tendons located in the same direction were more sensitive to the tip contact forces applied in that direction. This means that $F_{Ty+}$ and $F_{Ty-}$ were more sensitive to $F_y$ rather than $F_{Tx+}$ and $F_{Tx-}$. Therefore, if the contact force in each direction is estimated with two sub-estimators that both have half the number of neurons in the hidden layers of the single estimator, in our model, the number of neurons in the first hidden layer was 40, and the number of neurons in the second hidden layer was 50. This would reduce the computational resource consumption, such as the floating-point operations (FLOPs), as the number of cells per neural network layer would be halved with the manual decoupling of the sub-networks, which would result in a large reduction in the layer-to-layer multiplication operations (as they are replaced by two parallel sub-network calculations). If this manual decoupling method is used, the network structure in Fig. 5 can be changed to that shown in Fig. 9.

**Figure 10:**
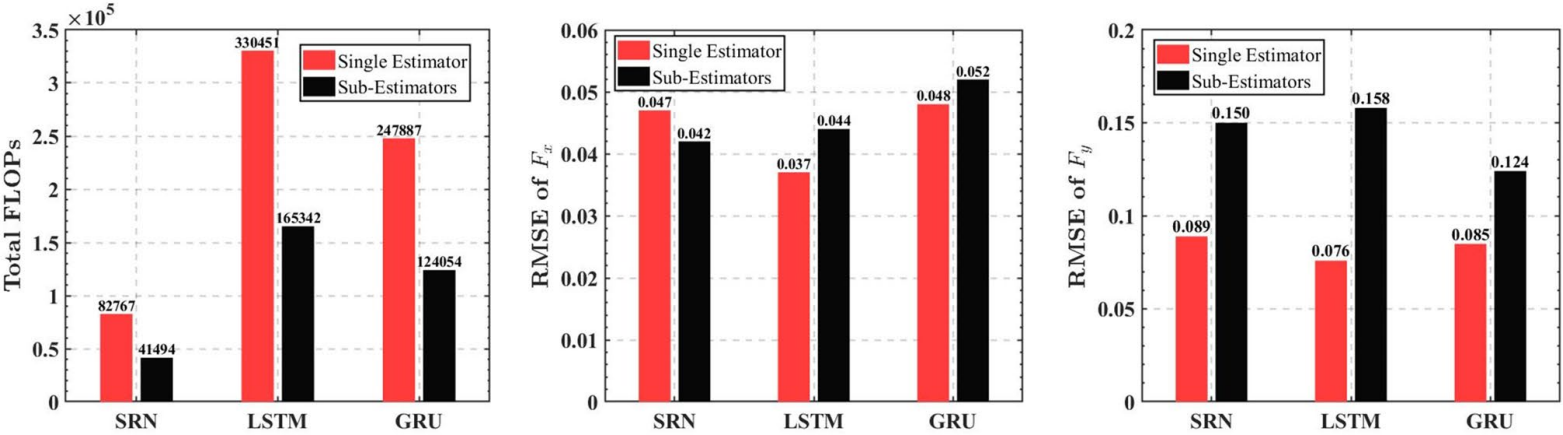
Performance comparisons between the two schemes.

However, we found there are still some disadvantages of this approach. Fig. 10 shows a comparison of the performance of the sub-estimators with that of one 2-DOF estimator. As we can see, the scheme with two sub-RNN estimators reduced the FLOPs by about $50\%$ compared to a single estimator. The sub-estimator for the *x*-direction did not differ much from the single-estimator in terms of accuracy, but the sub-estimator for the *y*-direction was less accurate than the single estimator. The reason for this condition can be explained by the following:

6 $$\begin{aligned} \begin{aligned} {\widehat{{F_y}}}&= g\left( {{q_y},{q_x},{F_{Ty + }},{F_{Ty - }},{F_{Tx + }},{F_{Tx - }}} \right) \\&= {g_y}\left( {{q_y},{F_{Ty + }},{F_{Ty - }}} \right) + {g_x}\left( {{q_x},{F_{Tx + }},{F_{Tx - }}} \right) \end{aligned} \end{aligned}$$

Ideally, the contact force $F_y$ would not cause a deformation of the two tendons in the *x*-direction, and $g_x$ should be equal to 0. However, owing to the existence of manufacturing and assembly errors, the tendon channels in the *x*-direction do not completely coincide with the *x*-direction of the contact force coordinates, resulting in $g_x$ not being equal to 0.

## Conclusions and future work

In this paper, we proposed a learning-based tip contact force estimation method for tendon-driven continuum manipulators, which can be more general and efficient for tendon-driven CMs compared to the method of building a complex static model. We also built a 3-DOF contact force acquisition platform based on the structure of the CM designed in our previous studies. After completing the data acquisition, we built four different ANN structures (MLP, SRN, LSTM, and GRU) as estimators with TensorFlow framework, and we also compared their accuracy and dynamic performances. We discovered that RNN-based estimators can effectively perform tip contact force estimation for CM that compared to MLP. Finally, we proposed a manually decoupled sub-estimator algorithm that can reduce the FLOPs by about 50% when compared to a single RNN-based estimator. However, due to manufacturing and assembly errors, this algorithm does not have to be as accurate as a single estimator method.

In future work, we will try to use the tip position of the CM collected by the electromagnetic sensors after being subjected to a contact force as a neural network output simultaneously with the tip contact force data to achieve the position feedback of the CM. An alternative is for the tip position to be used as the input data of the neural network to improve the tip contact force sensing accuracy. In this study, we used an RNN to fit a static model, which means the velocities of loading and unloading were ignored. So, in the future, we will try to design a more automated training data acquisition platform in order to attempt to fit the CM’s dynamical model to achieve a higher estimation accuracy. We will also try to integrate this algorithm into a robot-assisted surgery scene. What is more, the generalization ability across devices of this method is only theoretically possible and still needs to be validated in other types of robotic systems.
